# Acetylation State of Lysine 14 of Histone H3.3 Affects Mutant Huntingtin Induced Pathogenesis

**DOI:** 10.3390/ijms232315173

**Published:** 2022-12-02

**Authors:** Anikó Faragó, Nóra Zsindely, Anita Farkas, Alexandra Neller, Fruzsina Siági, Márton Richárd Szabó, Tamás Csont, László Bodai

**Affiliations:** 1 Department of Biochemistry and Molecular Biology, Faculty of Science and Informatics, University of Szeged, Közép fasor 52, H-6726 Szeged, Hungary; 2 Doctoral School in Biology, Faculty of Science and Informatics, University of Szeged, H-6726 Szeged, Hungary; 3 Department of Biochemistry, Albert Szent-Györgyi Medical School, University of Szeged, H-6720 Szeged, Hungary; 4 Interdisciplinary Centre of Excellence, University of Szeged, H-6720 Szeged, Hungary

**Keywords:** Huntington’s disease, neurodegeneration, epigenetics, histone, histone acetylation, *Drosophila*, animal model

## Abstract

Huntington’s Disease (HD) is a fatal neurodegenerative disorder caused by the expansion of a polyglutamine-coding CAG repeat in the *Huntingtin* gene. One of the main causes of neurodegeneration in HD is transcriptional dysregulation that, in part, is caused by the inhibition of histone acetyltransferase (HAT) enzymes. HD pathology can be alleviated by increasing the activity of specific HATs or by inhibiting histone deacetylase (HDAC) enzymes. To determine which histone’s post-translational modifications (PTMs) might play crucial roles in HD pathology, we investigated the phenotype-modifying effects of PTM mimetic mutations of variant histone H3.3 in a *Drosophila* model of HD. Specifically, we studied the mutations (K→Q: acetylated; K→R: non-modified; and K→M: methylated) of lysine residues K9, K14, and K27 of transgenic H3.3. In the case of H3.3K14Q modification, we observed the amelioration of all tested phenotypes (viability, longevity, neurodegeneration, motor activity, and circadian rhythm defects), while H3.3K14R had the opposite effect. H3.3K14Q expression prevented the negative effects of reduced *Gcn5* (a HAT acting on H3K14) on HD pathology, while it only partially hindered the positive effects of heterozygous *Sirt1* (an HDAC acting on H3K14). Thus, we conclude that the Gcn5-dependent acetylation of H3.3K14 might be an important epigenetic contributor to HD pathology.

## 1. Introduction

Huntington’s disease (HD, OMIM #143100) is a devastating, late-onset neurodegenerative disorder that primarily leads to the loss of medium spiny neurons in the striatum, but other areas of the brain are also affected [1]. HD is a dominantly inherited monogenic disease caused by mutations affecting a polymorphic CAG trinucleotide repeat in the first exon of the *Huntingtin* (*HTT,* HGNC:4851) gene. Mutant *HTT* alleles carry an expansion of the CAG repeat, which is translated to an elongated polyglutamine (polyQ) repeat close to the N-terminus of the huntingtin (Htt) protein. While short polyQ regions are not pathologic, polyQ stretches over 39 glutamines, inducing HD with full penetrance [1]. HD pathology is predominantly attributed to the gain-of-function effects of the mutation, although partial loss of normal Htt function might also play a role in the disease [1,2]. Similar mutations are responsible for pathogenesis in at least eight other neurodegenerative polyglutamine disorders [3]. Mutant Huntingtin (mHtt) is an aggregation-prone protein that forms nuclear and cytoplasmic aggregates that sequester cellular regulators [4]. Furthermore, soluble mHtt and/or its oligomers can bind to various proteins involved in key cellular processes, such as ribosome biogenesis, metabolism, intracellular transport, chromatin assembly, signal transduction, and transcription [5,6]. The effects that mHtt exerts on these processes might contribute to the multifaceted pathology of HD, which includes transcriptional alterations, impaired proteostasis, mitochondrial dysfunction, impaired molecular stress responses, and altered synaptic plasticity [4,7].

Transcriptional dysregulation is one of the major molecular pathomechanisms of HD that can be observed in both human neuronal tissues and model organisms [8,9,10]. In patient samples, similar gene expression changes were observed in affected brain regions, including the caudate nucleus, cerebellum, and parts of the frontal cortex [9], and the transcriptional alterations observed in human samples have been recapitulated in several mouse HD models as well [11]. Transcriptional changes preceded the manifestation of disease symptoms in several HD mouse models [12,13] suggesting that disturbed gene regulation is not a consequence, but a fundamental mechanism, of pathology. Chromatin-modifying mechanisms, including covalent post-translational modifications (PTM) of histone proteins, play a pivotal role in the regulation of transcription, and several lines of evidence point to epigenetic mechanisms in HD pathogenesis [14]. Accordingly, the striatal and hippocampal gene expression signatures of pre-symptomatic HD mice showed overlap with the transcriptional profiles of mice lacking specific histone-modifying enzymes, including the histone methyltransferases G9a (euchromatic histone-lysine N-methyltransferase 2, EHMT2), GLP (G9a-like protein, EMHT1) and EZH1/EZH2 (enhancer of zeste 1 and 2), and the histone acetyltransferase (HAT) CBP (CREB-binding protein) [12]. In symptomatic HD mice, the acetylation of histone H3 was found to be decreased at specific gene promoters in the absence of bulk changes in H3 acetylation [15], and the genome-wide analysis of striatal samples showed that the level of H3K9/K14 acetylation on gene sequences and the number of genes with H3 acetylation were both decreased [16].

Despite the long history of epigenetic studies in HD, it has still not been elucidated which specific histone PTMs play crucial roles in pathogenesis, although this knowledge would advance the design of therapeutic strategies. Previously, we investigated the effects of H3K27 methylation on mHtt-induced phenotypes using PTM mimetic constructs [17]. In the present study, we used the same approach to determine whether modifications of specific lysine residues of H3 that are targets of CBP and/or PCAF (P300/CBP-associated factor), HATs that were implicated in HD previously [18,19,20], influence HD pathology. To this end, we generated PTM mimetic *His3.3A* constructs with point mutations mimicking unmodified, acetylated, or methylated lysines at positions 9, 14, or 27 and tested them in a *Drosophila* HD model, which was based on *elav-GAL4*-driven neuron-specific expression of the first exon of human HTT with an elongated CAG repeat encoding for 120 glutamines (*HTTex1.Q120*) [21]. Our results show that the acetylation state of the K14 residue of the histone H3 tail has a profound effect on HD pathology, suggesting that it might be a potential therapeutic target.

## 2. Results

### 2.1. H3.3 PTM Mimetic Mutants Localize to Chromatin

**Figure 1:**
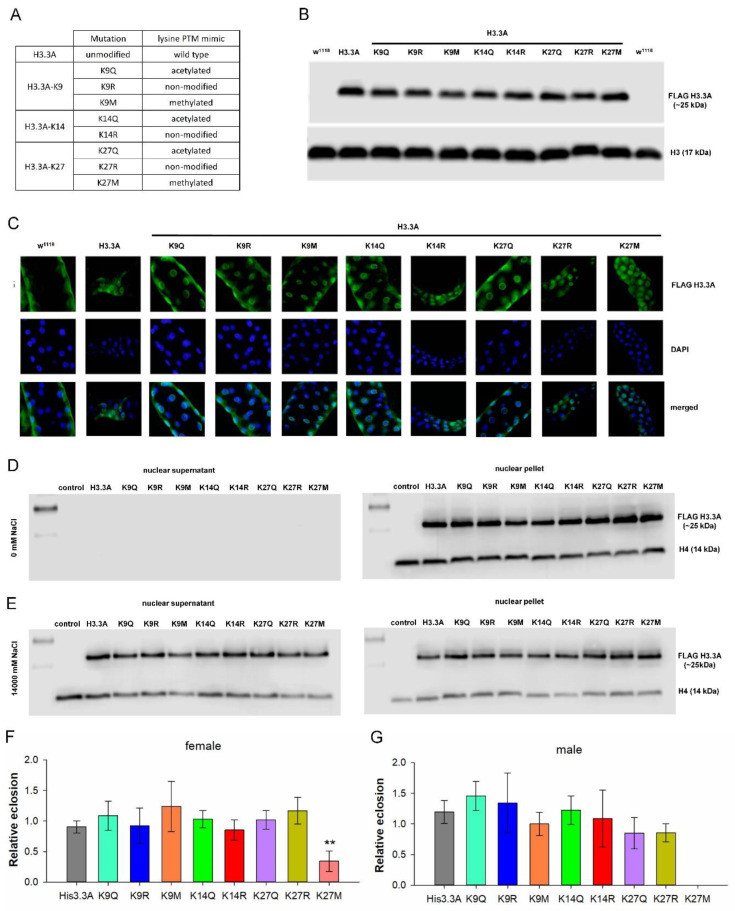
H3.3 PTM mimetic mutant histones are expressed at similar levels and show nuclear localization. Except for H3.3-K27M modification *elav-GAL4*-driven expression of H3.3-PTM, mimetic mutant histones do not affect the viability of wild-type flies. (**A**) List of generated transgenic flies mimicking lysine post-translational modifications of H3.3. Lysine mutated to glutamine (K→Q) mimics an acetylated state, lysine mutated to arginine (K→R) mimics a non-modified state, and lysine mutated to methionine (K→M) mimics a methylated state. (**B**) Immunoblot analysis of head samples of female flies overexpressing *His3.3A* or *His3.3A-PTM* transgenes using anti-FLAG antibody recognizing the C-terminal FLAG-tag. All transgenes produced full-length H3.3 proteins at similar levels. (**C**) Immunohistochemistry analysis of salivary glands of L3 larvae overexpressing *His3.3A* or *His3.3A-PTM* transgenes using anti-FLAG antibody. Mutant H3.3 histones are properly localized to nuclei. (**D**,**E**) Immunoblot analysis of histone salt elution experiments on head samples of male flies overexpressing *His3.3A* or *His3.3A-PTM* transgenes using anti-FLAG and anti-H4 antibodies. Without salt elution, histones were present at comparable levels in the nuclear pellet fraction, but could not be detected in the nuclear supernatant. In the presence of 1400 mM NaCl, H3.3 and H4 were partially eluted from chromatin and appeared both in the supernatant and pellet fractions. The similar levels in the eluted and chromatin-bound fractions indicate that the introduced point mutations do not alter the chromatin-binding affinity of H3.3. (**F**,**G**) Relative eclosion rate of *His3.3A-PTM*-expressing female and male flies. The overexpression of the *His3.3A* transgene variants does not decrease the viability of flies, except for *His3.3A-K27M*, where the eclosion rate of females is approximately 30% of the non-expressing controls, while males do not eclose. The bars show the ratio of eclosed flies, and error bars represent the standard error. *n* ≥ 90, ** *p* ≤ 0.01, ANOVA.

To investigate the effects of specific post-translational modifications of lysine residues of the N-terminal tail of H3 on HD phenotypes, first, we generated transgenic *Drosophila* strains (Figure 1A, Supplementary Figure S1A,B) carrying a FLAG-tag-labelled variant histone H3.3 expressing transgene (*UAS-His3.3A-FLAG*) or its PTM mimic mutant versions (henceforth *His3.3A-PTM*). In the *His3.3A-PTM* mutants, codons of specific lysine residues were changed to ones encoding glutamine, arginine, or methionine residues mimicking acetylated, unmodified, or methylated lysine, respectively. Specifically, lysine K9 was changed to glutamine (K9Q), arginine (K9R), or methionine (K9M), lysine K14 was changed to glutamine (K14Q) or arginine (K14R), and lysine K27 was changed to glutamine (K27Q) (Figure 1A). *His3.3A-K27R* and *His3.3A-K27M* stocks were generated using the same approach described previously [17].

All *His3.3A* transgenes were inserted into the same genomic position on the third chromosome (docking site ΦX-86Fb) by φC31 phage integrase-mediated site-specific integration [22] to provide a uniform genomic environment for transgene expression. All transgenic strains were verified to carry the desired mutations by Sanger sequencing.

For further validation steps, we investigated whether full-length transgenic H3.3-PTM proteins are produced, localized to the nucleus, and bound to chromatin, and whether their overexpression affects viability. To show that full-length proteins are produced by the various *His3.3A* transgenes, we performed immunoblot analysis on head samples of flies overexpressing *His3.3A* or *His3.3A-PTM* transgenes using anti-FLAG antibody recognizing the C-terminal FLAG-tag, and found that all transgenes produced full-length H3.3 proteins at similar levels (Figure 1B).

Next, we showed that both wild-type and mutant H3.3 histones localized to nuclei by immunohistochemistry with an anti-FLAG-specific antibody (Figure 1C). To demonstrate that transgenic H3.3 proteins were chromatin-bound, we isolated nuclei from the head lysates of transgenic flies and performed histone salt elution experiments. After optimizing the salt concentration for H3.3 elution from chromatin (Supplementary Figure S2), we performed nuclei isolation and histone salt elution of the head samples of *His3.3A* and *His3.3A-PTM*-overexpressing flies and non-expressing controls, followed by immunoblot analysis with anti-FLAG antibody to detect H3.3 and anti-H4 antibodies as the control. Our results indicated that, without salt elution, H3.3 and H4 were present in the nuclear pellet fraction containing chromatin, but could not be detected in the non-chromatin-bound nuclear supernatant (Figure 1D). In the presence of 1400 mM NaCl, H3.3 and H4 were partially eluted from chromatin and appeared both in the supernatant and pellet fractions (Figure 1E). Transgenic H3.3 and its various mutant forms were present at similar levels in the eluted and chromatin-bound fractions, indicating that the introduced point mutations did not alter the chromatin-binding affinity of H3.3.

To determine whether the overexpression of *His3.3A-PTM* transgenes caused neuronal toxicity, we tested the effect of their expression on viability using the *elav-GAL4* pan-neuronal diver. We found that the overexpression of the *His3.3A* transgene variants did not decrease the viability of flies, except for *His3.3A-K27M*, where the eclosion rate of females was approximately 30% (*p* = 5.6 × 10^−3^) of that of the non-expressing controls (Figure 1F), while males did not eclose (Figure 1G).

### 2.2. The Effects of H3.3-PTM Mutants on the Viability, Longevity, and Neurodegeneration of HD Flies

**Figure 2:**
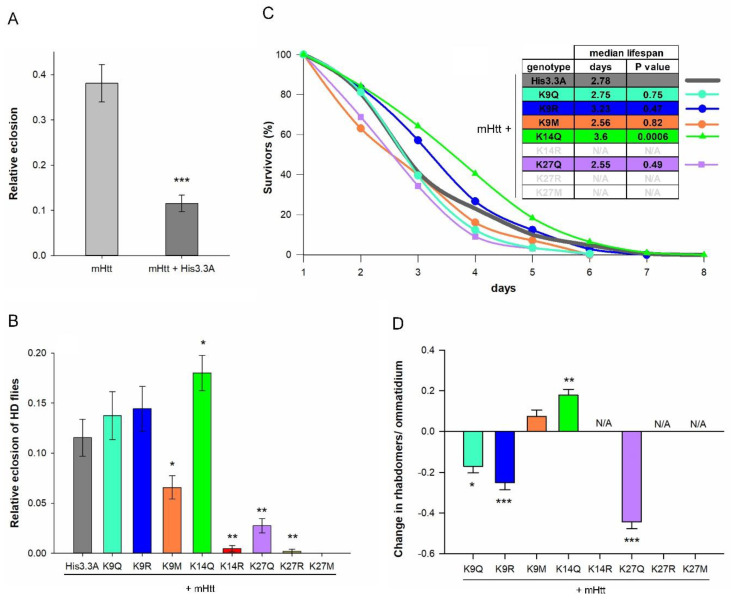
K14Q modification of H3.3 ameliorates HD phenotypes. (**A**) Relative eclosion rates of female flies expressing *mHtt* or co-expressing *mHtt + His3.3A* in the nervous system. The expression of wild-type *His3.3A* transgene decreased the eclosion rate of HD flies; thus, in all further studies, *mHtt + His3.3A co*-expressing flies served as controls. The bars show the ratio of eclosed flies and error bars represent standard error. *n* ≥ 1200, *** *p* ≤ 0.001, Student’s *t*-test. (**B**) Relative eclosion rates of *elav-GAL4* driven *mHtt + His3.3A* and *mHtt + His3.3A-PTM* expressing females. The eclosion rate of *His3.3A-K14Q*-expressing HD flies significantly increased, while the other modifications did not have an effect or worsened the phenotype compared with the control. The bars show the ratio of eclosed flies and the error bars represent the standard error. *n* ≥ 1200, * *p* ≤ 0.05, ** *p* ≤ 0.01, ANOVA. (**C**) Lifespan analysis of female flies co-expressing *mHtt + His3.3A* or *mHtt + His3.3A-PTM* in the nervous system. The median lifespan of *His3.3A-K14Q*-expressing HD flies was significantly longer, while the other modifications did not have a significant effect compared with the control. The graph shows the percentage of survivors as a function of the number of days after eclosion, *n* ≥ 150, Fisher’s exact test was used for statistical analysis of the median lifespan. (**D**) Pseudopupil assay of *elav-GAL4*-driven *mHtt + His3.3A-PTM*-expressing 2-day-old female flies. The expression of *His3.3A-K14Q* ameliorated neurodegeneration in HD flies, while the other modifications did not have an effect or worsened the phenotype compared with the control. The bars show the average difference in rhabdomere count compared to the *mHtt + His3.3A* expressing control and the error bars represent the standard error. *n* ≥ 10 eyes (≥30 ommatidia/eye), * *p* ≤ 0.05, ** *p* ≤ 0.01, *** *p* ≤ 0.001, ANOVA.

After validation, we performed genetic interaction tests to evaluate the effects of H3.3-PTM mutants on mHtt-induced phenotypes. To this end, we co-expressed *His3.3A* or *His3.3A-PTM* transgenes with an *mHtt* transgene containing the first exon of mutant human *HTT* with 120 glutamines (*UAS-HTTex1.Q120)* using the pan-neuronal *elav-GAL4* driver. First, we tested the effect of *His3.3A-PTM* transgenes on the viability of HD flies. Neuronal mHtt expression is toxic to flies, resulting in decreased eclosion rates [23]. To our surprise, the expression of wild-type *His3.3A* transgene decreased the eclosion rate of HD flies even further, underlining the sensitivity of the HD model to epigenetic effects (Figure 2A). Therefore, in all further experiments, we compared the experimental categories to controls co-expressing *His3.3A* and *Httex1.Q120*.

By analyzing the effects of *His3.3A-PTM* transgenes, we found that the eclosion rate of HD flies expressing *His3.3A-K14Q* was significantly higher (*p* = 2.2028 × 10^−2^) compared with *His3.3A*-expressing HD flies, but other PTM mimetic mutants did not have similar positive effects (Figure 2B). The eclosion rates of *His3.3A-K9Q*- or *His3.3A-K9R*-expressing HD flies were similar, while those of *His3.3A-K9M*, *His3.3A-K14R*, *His3.3A-K27Q*, or *His3.3A-K27R*-expressing flies were significantly lower (*p* = 2.2606 × 10^−3^, *p* = 1.0053 × 10^−3^, *p* = 7.6515 × 10^−3^, and *p* = 1.0053 × 10^−3^, respectively) compared with the control. *His3.3A-K27M* could not be tested due to its toxic effects if driven by *elav-GAL4*.

The flies collected during eclosion rate testing were also used for longevity analysis. We found that the median lifespan of *His3.3A-K14Q*-expressing HD flies was significantly longer (3.6 days, *p* = 6 × 10^−4^) compared with the non-modified *His3.3A*-expressing HD flies (2.78 days), while the median lifespan of *His3.3A-K9Q* (2.75 days), *His3.3A-K9R* (3.23 days), *His3.3A-K9M* (2.56 days), and *His3.3A-K27Q* (2.55 days) did not deviate significantly from that of the control (Figure 2C).

Finally, we determined the extent of neurodegeneration of *His3.3A-PTM*- or *His3.3A*-expressing HD flies using the pseudopupil assay to visualize photoreceptor neurons in the eyes of 2-day-old females. The expression of *His3.3A-K14Q* significantly suppressed neurodegeneration, measured as the average number of rhabdomeres per ommatidium (*p* = 5.73 × 10^−3^); contrarily, the expressions of *His3.3A-K9Q*, *His3.3A-K9R*, and *His3.3A-K27Q* significantly aggravated neurodegeneration (*p* = 1.44 × 10^−2^, *p* = 2.97 × 10^−4^, and *p* = 1.5 × 10^−6^, respectively). In HD flies expressing *His3.3A-K9M*, there was no significant difference in neurodegeneration (Figure 2D). We could not perform longevity and pseudopupil assays in the case of *His3.3A-K14R*, *His3.3A-K27R*, and *His3.3A-K27M*-expressing HD flies due to the insufficient number of eclosing adults.

As, in the genetic interaction experiments, both the *mHtt* transgene and the *His3.3A* or *His3.3A-PTM* transgenes were expressed under the control of the *elav-GAL4*/*UAS* system, there was a possibility for competence between the two UAS transgenes for GAL4 binding. To demonstrate that the observed phenotypes did not result from the reduced expression of either the *mHtt* or *His3.3A-PTM* transgenes, we performed RT-qPCR measurements to determine the gene expression levels of the transgenes. *mHtt* transgene expression did not change significantly if co-expressed with *His3.3A-PTM* transgenes (Supplementary Figure S3A). Similarly, there was no significant difference in the expression levels of *His3.3A-PTM* transgenes (Supplementary Figure S3B). Thus, the observed phenotypes were not consequences of altered *mHtt* or *His3.3A-PTM* expression, but rather of the specific lysine modifications of H3.3.

### 2.3. Adult Neuronal Expression of H3.3K14Q Improves Longevity and Mitigates Motor Dysfunction and Sleep Defects of HD Flies

The severe effects of co-expression of *mHtt* and *His3.3A* or its mutant variants from embryogenesis with the *elav-GAL4* driver did not allow behavioral analysis to be performed. Therefore, we decided to induce their expression in adult neurons using *elav-GAL4* combined with the *tub-GAL80^ts^* transgene that expresses a temperature-sensitive allele of GAL80, a negative regulator of GAL4 [24]. In order to reduce the severe effects of H3.3 overexpression in HD flies, we also introduced a heterozygous deletion of endogenous *His3.3A* (*His3.3A^KO^*) in these studies to reduce the total H3.3 load.

**Figure 3:**
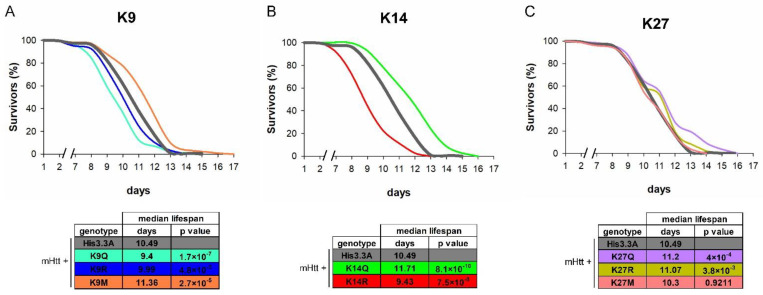
Adult expressions of *K9M, K14Q, K27Q, or* K27R mutant H3.3-elongated median lifespans of HD flies. Lifespan analysis of male flies co-expressing *mHtt + His3.3A-PTM* since eclosion in the nervous system under the influence of *elav-GAL4* and *tubGAL80^ts^* over a heterozygous *His3.3A^KO^* background. (**A**) Expression of *His3.3A-K9M* increased the median lifespan of HD flies, while the expression of *His3.3A-K9Q* and *His3.3A-K9R* shortened it. (**B**) *His3.3A-K14Q* increased median lifespan, while *His3.3A-K14R* had the opposite effect. (**C**) Expression of *His3.3A-K27Q* and *His3.3A-K27R* transgenes increased median lifespan, while *His3.3A-K27M* had no effect. Graphs show the percentage of survivors as a function of the number of days after eclosion, *n* ≥ 200. Fisher’s exact test was used for statistical analysis of median lifespans.

First, we analyzed the lifespan of HD flies overexpressing H3.3 with modified K9, K14, or K27 (the effect of K27R and K27M on the longevity of HD flies was published previously with very similar results [17]). We found that the median lifespans of *His3.3A-K9M* (11.36 days, *p* = 2.7 × 10^−5^), *His3.3A-K14Q* (11.71 days, *p* = 8.1 × 10^−10^), *His3.3A-K27Q* (11.2 days; *p* = 4 × 10^−4^), and *His3.3A-K27R* (11.07 days; *p* = 3.8 × 10^−3^)-expressing male HD flies were significantly longer than those of *His3.3A*-expressing controls (10.49 days) (Figure 3A–C).

In contrast, the median lifespans of *His3.3A-K9Q* (9.4 days, *p* = 1.7 × 10^−7^), *His3.3A-K9R* (9.99 days, *p* = 4.8 × 10^−3^), or *His3.3A-K14R* (9.43 days, *p* = 7.5 × 10^−8^)-expressing males were shorter, while that of *His3.3A-K27M*-expressing males did not change (Figure 3A–C).

**Figure 4:**
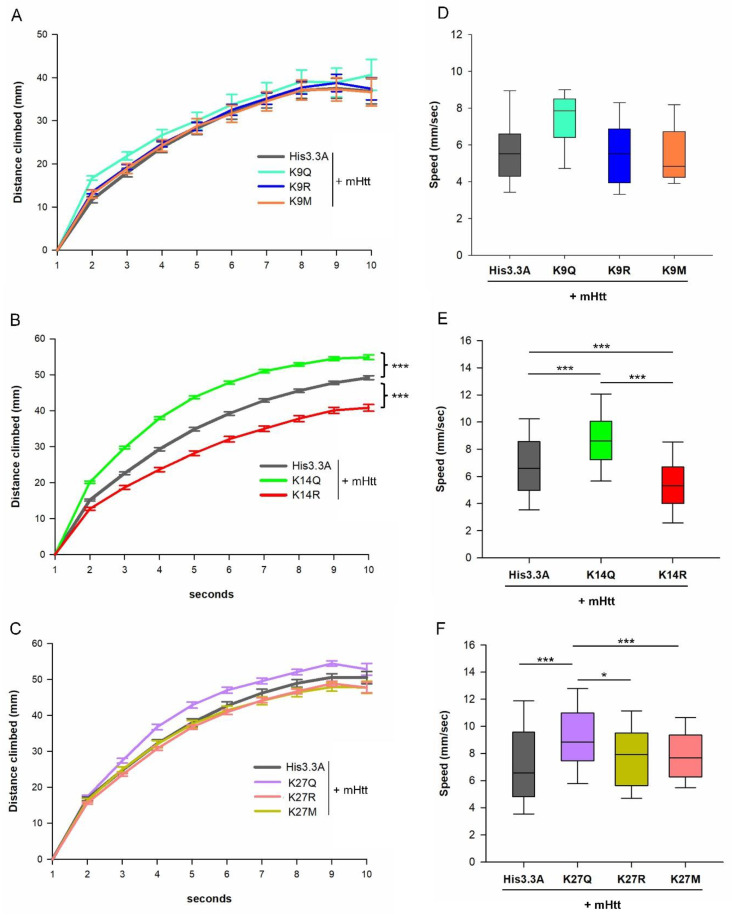
Adult expression of K14Q mutant H3.3 ameliorates, while K14R mutant exacerbates motor phenotypes in HD flies. (**A**–**C**) Vertical distances climbed during a 10 s time period by male flies co-expressing *mHtt + His3.3A-PTM* in the adult nervous system under the influence of *elav-GAL4*; *tubGAL80^ts^* with heterozygous *His3.3A^KO^* background. Graphs show the average climbing distance in mm and the error bars represent standard error; a two-way ANOVA was used for the statistical analysis of climbing curves. (**B**) In *His3.3A-K14Q*-expressing flies, climbing ability improved (genotype/height interaction *p* = 3.79 × 10^−10^) compared with the unmodified *His3.3A* expressing controls, while that of *His3.3A-K14R*-expressing flies was significantly worse (genotype/height interaction *p* = 3.33 × 10^−16^). No significant differences were observed in HD flies carrying His3.3A transgenes with mutations affecting the K9 (**A**) or K27 (**C**) residues. (**D**–**F**) Climbing speed between the 2nd and 3rd seconds of male flies co-expressing *mHtt + His3.3A-PTM* in the adult nervous system under the influence of *elav-GAL4*; *tubGAL80^ts^* with heterozygous *His3.3A^KO^* background. (**D**) Point mutations of *His3.3A* affecting lysine K9 do not affect the climbing speed compared with the control. (**E**) Climbing speed of *His3.3A-K14Q*-expressing flies improved, while *His3.3A-K14R*-expressing flies climbed slower compared with the control. (**F**) Climbing speed of *His3.3A-K27Q*-expressing flies also improved compared with the control. Boxplots show the distribution (first quartile, median, third quartile, and 10th and 90th percentiles as whiskers). *n* ≥ 60, * *p* ≤ 0.05, *** *p* ≤ 0.001, ANOVA.

Next, we performed motor activity measurements on the eclosed male HD flies expressing *His3.3A-PTM* transgenes with a heterozygous *His3.3A^KO^* background. Motor activity was determined by measuring the vertical distance climbed in a 10 s timeframe and by calculating the speed of climbing between the second and third seconds. The distance climbed in 10 s was significantly increased in *His3.3A-K14Q*-expressing flies (genotype/height interaction *p* = 3.79 × 10^−10^) compared with the wild-type *His3.3A*-expressing controls, while that of *His3.3A-K14R*-expressing flies was significantly decreased (genotype/height interaction *p* = 3.33 × 10^−16^) (Figure 4B). In the case of *His3.3A-K9Q, -K9R, -K9M*, and *His3.3A-K27Q, -K27R, and -K27M* mutants, we did not detect significant differences in the climbing distance (Figure 4A,C).

By analyzing climbing speed, we found that *His3.3A-K14Q*-expressing flies climbed significantly faster (8.6877 mm/s; *p* = 1.01 × 10^−3^), while *His3.3A-K14R* expressing flies climbed significantly slower (5.4045 mm/s; *p* = 1.01 × 10^−3^) than the unmodified *His3.3A*-expressing controls (6.7928 mm/s) (Figure 4E). Furthermore, *His3.3A-K27Q*-expressing flies also climbed significantly faster (9.0840 mm/s; *p* = 1.005 × 10^−3^) compared with control flies (7.2163 mm/s) (Figure 4F), while we did not detect significant differences in climbing speed in the case of *His3.3A-K9Q*, *-K9R*, and *-K9M* or *His3.3A-K27R* and *-K27M* mutants (Figure 4D,F).

**Figure 5:**
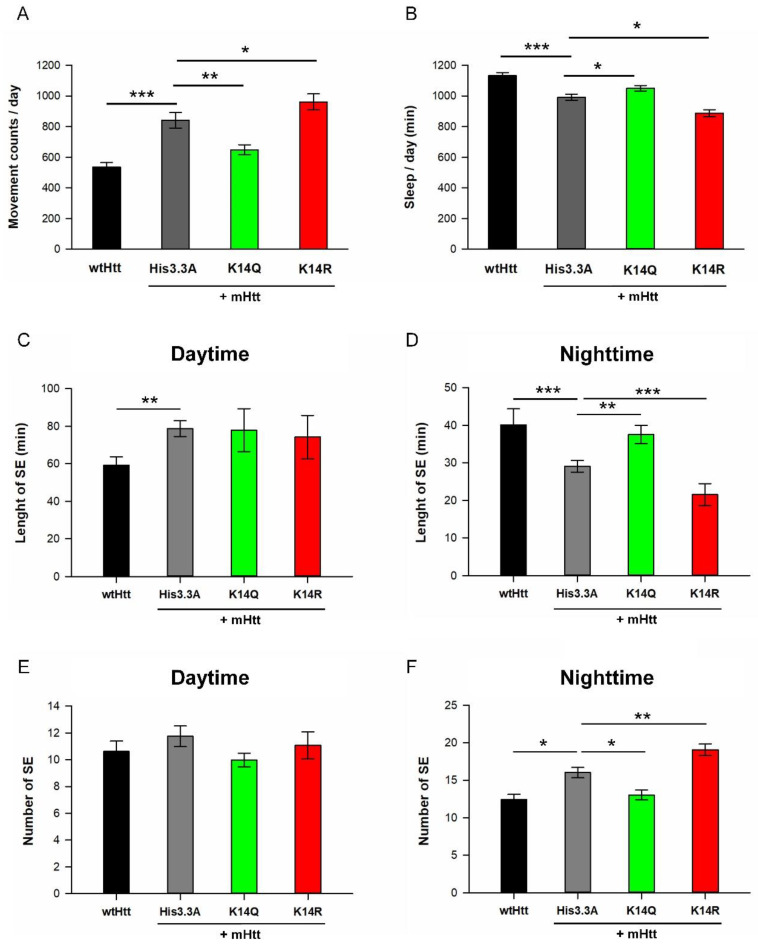
Adult expression of K14Q mutant H3.3 suppressed disturbed daily activity and sleep phenotypes in HD flies, while expression of the K14R mutant enhanced them. (**A**) Total daily movement counts of male flies expressing *wtHtt* or co-expressing *mHtt + His3.3A* transgenes in the adult nervous system under the influence of *elav-GAL4*; *tubGAL80^ts^* with heterozygous *His3.3A^KO^* background. Co-expression of mHtt and *His3.3A* leads to hyperactivity (increased movement counts) compared with flies expressing Huntingtin with 25 glutamines (*wtHtt*). Expression of *His3.3A-K14Q* suppressed hyperactivity of HD flies compared with *His3.3A*-expressing control flies, while expression of *His3.3A-K14R* enhanced it. The graph shows the average of the total number of movements during a 24 h time period; the error bars represent the standard error. (**B**) Total amount of daily sleep of male flies expressing *wtHtt* or co-expressing *mHtt + His3.3A* transgenes in the adult nervous system under the influence of *elav-GAL4*; *tubGAL80^ts^* with heterozygous *His3.3A^KO^* background. Control flies co-expressing mHtt and *His3.3A* slept significantly less than *wtHtt*-expressing flies. *His3.3A-K14Q*-expressing HD flies slept more, while *His3.3A-K14R*-expressing HD flies spent less time asleep compared with *His3.3A*-expressing control flies. The graph shows the average of the total amount of sleep in minutes during a 24 h time period and the error bars represent standard error. (**C**,**D**) Average length of sleep episodes during the daytime (**C**) and nighttime (**D**) for male flies co-expressing *mHtt + His3.3A-PTM* transgenes in the adult nervous system under the influence of *elav-GAL4*; *tubGAL80^ts^* with heterozygous *His3.3A^KO^* background. (**C**) Co-expression of *mHtt* + *His3.3A* transgenes increased the average length of daytime sleep episodes of male flies compared with *wtHtt* males, irrespective of the mutations of the K14 residue. (**D**) During the nighttime, the length of sleep episodes decreased in males co-expressing *mHtt* + *His3.3A* compared with those expressing *wtHtt*. This phenotype was suppressed by the K14Q mutation, while it was exacerbated by the K14R mutation of the *His3.3A* transgene. (**E**,**F**) Average number of sleep episodes during the daytime (**E**) and nighttime (**F**) of male flies co-expressing *mHtt + His3.3A-PTM* transgenes in the adult nervous system under the influence of *elav-GAL4*; *tubGAL80^ts^* with heterozygous *His3.3A^KO^* background. (**E**) During the daytime, the average number of sleep episodes of males co-expressing *mHtt* + *His3.3A* transgenes was similar to that of males expressing *wtHtt*. (**F**) During the nighttime, the average number of sleep episodes of males co-expressing *mHtt* + *His3.3A* in the adult nervous system was increased compared with males expressing *wtHtt*. This effect was suppressed by the K14Q mutation, while it was enhanced by the K14R mutation of the *His3.3A* transgene. *n* ≥ 24, * *p* ≤ 0.05, ** *p* ≤ 0.01, *** *p* ≤ 0.001, ANOVA.

Finally, we investigated the effects of H3.3 mutants on sleep defects induced by mHtt. We previously showed that sleep abnormalities observed in HD patients can be modeled in *Drosophila* [25]. Sleep defects in the HD model are characterized by reduced overall sleep, fragmented sleep, prolonged sleep-onset latency, and, consequently, increased daily activity. We performed daily activity measurements and sleep analysis on *His3.3A* or *His3.3A-PTM*-expressing male HD flies with a heterozygous *His3.3A^KO^* background. Consistent with our previous findings [25], male flies co-expressing *HTTex1.Q120* and *His3.3A* showed hyperactivity (*p* = 1.52 × 10^−7^) (Figure 5A) and spent less time asleep (*p* = 5.98 × 10^−6^) (Figure 5B, Supplementary Figure S4A) compared with *HTTex1.Q25*-expressing healthy flies. We found that the expression of *His3.3A-K14Q* significantly suppressed disturbed daily activity and sleep phenotypes in HD flies, while that of *His3.3A-K14R* significantly enhanced them. The total daily movement of *His3.3A-K14Q*-expressing HD males (649 ± 33 movement counts; *p* = 0.006) was significantly lower, while that of *His3.3A-K14R*-expressing HD males was significantly higher (961 ± 52 movement counts; *p* = 0.001) than that pf *His3.3A*-expressing HD controls (841 ± 51 movement counts) (Figure 5A). The length of the total daily sleep of *His3.3A-K14Q*-expressing HD flies significantly increased (1050 ± 18 min, *p* = 0.006), while that of *His3.3A-K14R*-expressing HD flies significantly decreased (898 ± 24 min, *p* = 0.001) compared with the *His3.3A*-expressing HD controls (992 ± 20 min) (Figure 5B, Supplementary Figure S4A). Previously, we found that *Httex1.Q120*-expressing HD flies showed sleep fragmentation phenotypes in both the daytime and nighttime: the length of sleep episodes was reduced, while their number was increased [25]. In the case of male flies co-expressing *HTTex1.Q120* and *His3.3A*, we only detected this pattern during nighttime sleep (Figure 5C–F). The expression of *His3.3A* transgenes in HD flies increased the average length of daytime sleep episodes compared with healthy flies, irrespective of the mutations of the K14 residue (Figure 5C). However, in *His3.3A*-expressing HD flies, we observed a significant decrease in the average length of nighttime sleep episodes (29 ± 2; *p* = 3.27 × 10^−5^) compared with healthy controls (40 ± 4). The expression of *His3.3A-K14Q* significantly increased (38 ± 2; *p* = 1.27 × 10^−3^) the average length of nighttime sleep episodes compared with *His3.3A*-expressing HD control flies, while the expression of *His3.3A-K14R* significantly decreased it (22 ± 3; *p* = 4.83 × 10^−4^) (Figure 5D). During the daytime, there was no significant difference in the number of sleep episodes of *His3.3A, His3.3A-K14Q*, or *His3.3A-K14R*-expressing HD flies compared with healthy controls (Figure 5E). However, during the nighttime in the case of *His3.3A*-expressing HD flies, we observed a significant increase in the average number of sleep episodes (16 ± 1; *p* = 0.028) compared with healthy flies (12 ± 1). The expression of *His3.3A-K14Q* significantly decreased (13 ± 1; *p* = 0.025) the number of sleep episodes compared with *His3.3A*-expressing HD flies, while the expression of *His3.3A-K14R* significantly increased it (19 ± 1; *p* = 8.12 × 10^−3^) (Figure 5F). Consistent with our previous findings [25], the activity index—movement count normalized to the time spent awake—of male flies co-expressing *HTTex1.Q120* and *His3.3A* was similar to that of *HTTex1.Q25*-expressing healthy flies, indicating that the observed hyperactivity was the consequence of reduced sleep, instead of increased motor activity. The comparison of activity indexes showed no significant differences in male flies co-expressing *HTTex1.Q120* with either *His3.3A*, *His3.3A-K14Q*, or *His3.3A-K14R* (Supplementary Figure S4B). We could not detect significant differences in sleep-onset latency—the time between lights ON/OFF and the start of the first sleep episode—in male flies expressing *HTTex1.Q25* or co-expressing *HTTex1.Q120* and *His3.3A*, *His3.3A-K14Q*, or *His3.3A-K14R* (Supplementary Figure S4C). In the case of *His3.3A-K9* and *His3.3A-K27* mutants, we did not detect significant differences in any aspects of the sleep rhythm (Supplementary Figure S5).

### 2.4. Negative Effects of Reduced GCN5 Are Averted in H3.3-K14Q-Expressing HD Flies

Having seen that the acetylation–mimetic modification of H3.3K14 ameliorates HD pathology, while its unmodifiable state makes it more severe, we aimed to investigate the functional link between H3K14 modification and histone-modifying enzymes in the HD model. We tested genetic interactions of the *His3.3A-K14Q* transgene with loss-of-function mutations of the histone acetyltransferase *Gcn5* and the histone deacetylase *Sirt1* in the HD model with a *His3.3A^KO^* heterozygous background. Similar genetic interaction tests were not performed with *His3.3A-K14R* transgene-overexpressing HD flies, as they did not eclose.

**Figure 6:**
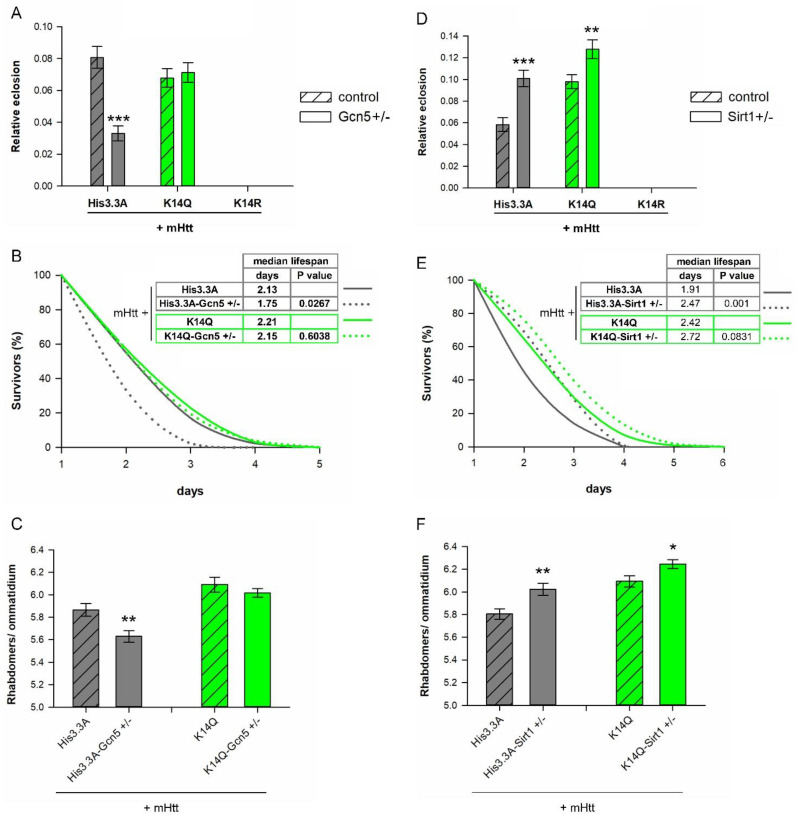
Heterozygous loss of *Gcn5* led to enhanced disease phenotypes in female flies expressing mHtt and wild-type H3.3, while it did not affect the phenotypes of females co-expressing mHtt and H3.3K14Q. (**A**) Relative eclosion rate of flies co-expressing *mHtt* + *His3.3A* or *mHtt* + *His3.3A-K14Q/R* in the nervous system and wild-type or heterozygous for a loss-of-function allele of *Gcn5*. The partial loss of *Gcn5* led to a reduced eclosion rate of wild-type *His3.3A* co-expressing HD flies, while, in the case of *His3.3A-K14Q* co-expression, there was no difference. As seen previously, *His3.3A-K14R*-expressing HD flies did not eclose. The bars show the ratio of eclosed flies and the error bars represent standard error. *n* ≥ 1800, *** *p* ≤ 0.001, Student’s *t*-test. (**B**) Longevity analysis of flies co-expressing *mHtt* + *His3.3A* or *mHtt* + *His3.3A-K14Q* in the nervous system and wild-type or heterozygous for *Gcn5*. Partial loss of *Gcn5* significantly decreased the median lifespan of unmodified *His3.3A*-expressing HD flies, while in the case of *His3.3A-K14Q*-expressing flies, *Gcn5* did not have an effect. The graph shows the percentage of survivors as a function of the number of days after eclosion, *n* ≥ 100; Fisher’s exact test was used for the statistical analysis of the median lifespan. (**C**) Pseudopupil assay of flies co-expressing *mHtt* + *His3.3A* or *mHtt* + *His3.3A-K14Q* in the nervous system and wild-type or heterozygous for *Gcn5*. Partial loss of Gcn5 significantly enhanced neurodegeneration in the eyes of unmodified *His3.3A*-expressing HD flies, while in the case of *His3.3A-K14Q*-expressing flies, it did not have an effect. The bars show the average number of rhabdomeres per ommatidium and the error bars represent the standard error. *n* ≥ 10 eyes (≥30 ommatidia/eye), ** *p* ≤ 0.01, Student’s *t*-test. (**D**) Relative eclosion rate of flies co-expressing *mHtt* + *His3.3A* or *mHtt* + *His3.3A-K14Q/R* in the nervous system and wild-type or heterozygous for *Sirt1*. Partial loss of *Sirt1* improved the eclosion rate of HD flies expressing either unmodified *His3.3A* or *His3.3A-K14Q* compared with their respective controls. *His3.3A-K14R*-expressing HD flies did not eclose. The bars show the ratio of eclosed flies and the error bars represent the standard error. *n* ≥ 1800, *** *p* ≤ 0.001, Student’s t-test. (**E**) Longevity of flies co-expressing *mHtt* + *His3.3A* or *mHtt* + *His3.3A-K14Q* in the nervous system and wild-type or heterozygous for *Sirt1*. Partial loss of *Sirt1* significantly increased the median lifespan of unmodified *His3.3A*-expressing HD flies, while it did not affect the median lifespan of *His3.3A-K14Q*-expressing HD flies. The graph shows the percentage of survivors as a function of the number of days after eclosion, *n* ≥ 100. Fisher’s exact test was used for the statistical analysis of the median lifespan. (**F**) Pseudopupil assay of flies co-expressing *mHtt* + *His3.3A* or *mHtt* + *His3.3A-K14Q* in the nervous system and wild-type or heterozygous for *Sirt1*. Partial loss of *Sirt1* significantly ameliorated neurodegeneration in the eyes of HD flies expressing either unmodified *His3.3A* or *His3.3A-K14Q* compared with their respective controls. The bars show the average number of rhabdomeres per ommatidium and the error bars represent standard error. *N* ≥ 10 eyes (≥30 ommatidia/eye), * *p* ≤ 0.05, ** *p* ≤ 0.01, Student’s *t*-test.

First, we tested *Gcn5* (single ortholog of human GCN5 and PCAF), a HAT that primarily acetylates the H3K14 residue (among other targets, including, H3K9, H3K18, H3K23, H3K27, and H3K36) [26]. Previously, mHtt was shown to bind to human PCAF in vitro [18] and, in flies, reduce *Gcn5*, leading to more pronounced mHtt toxicity [27]. We found that the heterozygous loss of *Gcn5* in flies expressing *mHtt* and wild-type *His3.3A* in the nervous system led to a reduced eclosion rate (0.0331 ± 0.005 vs. 0.0807 ± 0.007 of control, *p* = 3.28 × 10^−8^, Figure 6A), reduced longevity (median lifespan: 1.75 days vs. 2.13 days of controls, *p* = 0.0267, Figure 6B), and increased neurodegeneration (rhabdomeres/ommatidia: 5.63 vs. 5.86 in controls, *p* = 0.0067, Figure 6C) compared with control flies co-expressing *mHtt* and *His3.3A*, but wild-type for *Gcn5*. In contrast, in *His3.3A-K14Q*-expressing HD flies, the heterozygous loss of *Gcn5* did not lead to significant differences in either of these phenotypes (Figure 6A–C). Thus, our results indicate that, regarding the disease-modifying effect of *Gcn5*, the H3.3K14Q modification is epistatic over *Gcn5* and suggest that the involvement of GCN5 in HD pathology can be primarily attributed to the reduced acetylation of the H3K14 residue.

Next, we tested *Sirt1*/*Sir2* (ortholog of human SIRT1), a sirtuin-type histone deacetylase that modifies mHtt pathogenesis in several HD models [28]. Sirtuin enzymes deacetylate both histones and non-histone proteins, with H3K14 being one of the histone targets of SIRT1 [29]. As expected, the heterozygous loss of *Sirt1* in flies expressing *mHtt* and wild-type *His3.3A* led to a significantly improved eclosion rate (0.1009 ± 0.007 vs. 0.0584 ± 0.006 of control, *p* = 3.43 × 10^−5^ *t*-test, Figure 6D) and longevity (median lifespan: 2.47 days vs. 1.91 days of controls, *p* = 0.001, Figure 6E) and reduced neurodegeneration (rhabdomeres/ommatidia: 6.02 vs. 5.81 in controls, *p* = 0.00794, Figure 6F) compared with control flies co-expressing *mHtt* and *His3.3A*, but wild-type for *Sirt1*. In the case of HD flies expressing *His3.3A-K14Q*, the heterozygous loss of *Sirt1* resulted in a statistically significant improvement in the eclosion rate (0.1278 ± 0.008 vs. 0.0980 ± 0.006 of control, *p* = 0.0058, Figure 6D). There was no significant difference in longevity (Figure 6E); however, we observed significantly decreased neurodegeneration (rhabdomeres/ommatidia:6.24 vs. 6.09 in controls; *p* = 0.0298, Figure 6F) compared with control flies co-expressing *mHtt* and *His3.3A-K14Q*, but wild-type for *Sirt1*. Thus, these data suggest that the positive effects of reduced *Sirt1* levels in the fly HD model are only partially mediated by modifying the acetylation state of the H3K14 residue.

## 3. Discussion

Besides forming abnormal intracellular protein species, including oligomers, fibrils, and large aggregates, mHtt protein forms atypical interactions with other proteins, thereby impeding their function. One group of interacting partners is the HAT enzymes that modify the chromatin structure and influence gene transcription [18]. HATs acetylate the lysine residues of histone proteins by transferring an acetyl group from acetyl-CoA to the ε-amino group and neutralizing its charge. As a consequence, the chromatin structure is transformed into a more relaxed, accessible state that is associated with higher levels of gene transcription. Several studies have indicated that the Htt protein binds to the HATs CREB-binding protein (CBP/KAT3A) and P300/CBP-associated factor (PCAF/KAT2B), and that the interaction of these enzymes with mHtt leads to the inhibition of their catalytic activity and/or their degradation [18,19,30]. Furthermore, a recent study indicated that the histone acetyltransferase Tat interactive protein 60 kDa (Tip60 and KAT5) might be also involved in HD pathogenesis, as mHtt expression leads to diminished Tip60 levels and reduced histone H4 acetylation at its target genes in *Drosophila* [31]. The disturbed acetylation balance and consequent dysregulation of gene expression caused by the loss of function of specific HATs are thought to be responsible for some of the detrimental cellular effects observed in HD pathogenesis. This hypothesis is supported by experiments demonstrating that increasing the level of specific HATs [20,31,32] or inhibiting the histone deacetylase activity can alleviate mHtt-induced symptoms [33,34,35], supposedly by restoring the acetylation balance and transcriptional regulation. It is still not known, however, which histone acetylation sites are most important in terms of HD pathogenesis and possible therapeutical applications. Additionally, the increased acetylation of histone H4 and H3 that was observed in specific brain regions of HD patients raises concern about the application of generic HDAC inhibitors [36].

Therefore, in this study, we investigated the effects of specific histone acetylation marks in an HD model using PTM mimetic mutations of the K9, K14, and K27 residues of the variant histone H3.3. We selected the application of *His3.3A* transgenes to introduce a histone variant that is incorporated into active genes and to avoid developmental defects that might occur when modifying canonical H3 in the histone gene cluster. We generated missense mutations of the K9, K14, and K27 lysine residues that mimic acetylated lysine (K to Q), non-modified lysine (K to R), or methylated lysine (K to M). By analyzing the viability, longevity, neurodegeneration, motor abilities, and daily activity of HD flies co-expressing different *His3.3A* transgenes, we found that modifying K9 and K27 lysine had no remarkable effect on HD pathogenesis, the symptoms did not change significantly, or the different assays showed controversial results. However, the expression of *His3.3A* with specific PTM mimetic forms of the K14 residue led to consistent results in different assays. Expressing the acetylation mimicking H3.3K14Q ameliorated the phenotypes of HD flies in every assay performed (increased eclosion rate, delayed 50% mortality, improved climbing ability, and reduced neurodegeneration and sleep defects), while expressing the non-modified lysine mimicking H3.3K14R made all of the above-mentioned phenotypes more severe.

The acetylation of the H3K14 residue is involved in various chromatin-related processes, including the regulation of chromatin structure [37], transcription [38,39], and DNA repair [40]. This epigenetic mark has been frequently investigated and discussed in parallel with the acetylation of the H3K9 residue; however, due to the imperfect selectivity of the antibodies used in some of these studies, the effects of the two modifications often could not be differentiated. It is also important to note that, as the N-terminal 30 amino acid residues of *Drosophila* H3 and H3.3 and mammalian H3.1, H3.2, and H3.3 are identical, antibodies specific for the N-terminal PTMs of H3 also recognize the modified forms of all of these variant proteins. A detailed ChIP-seq study performed in mouse embryonic stem cells using highly specific antibodies able to specifically recognize acetyl-H3K9 or acetyl-H3K14 showed that, although the localization of the two marks frequently overlaps, it is not identical. Both PTM marks were shown to be present at promoters, exons, introns, and distal intergenic regions alike. The levels of H3K9 and H3K14 acetylation showed a strong correlation at promoters with bimodal distribution around the transcriptional start sites (TSSs), and the level of acetyl-H3K14 correlated with the level of gene expression on active promoters, emphasizing its role in gene regulation. Acetyl-H3K14 is also present on poised and active bivalent promoters and on enhancer elements, and is especially enriched on active enhancers. Although, similarly to acetyl-H3K9, acetyl-H3K14 frequently occurs together with active epigenetic marks, in contrast to acetyl-H3K9, it also shows a strong correlation with inactive histone PTM marks and marks inactive promoters [38]. These data suggest that acetyl-H3K14 might have a role in maintaining the poised state of genes for future activation. Accordingly, several studies indicated that H3K14 acetylation is involved in gene activation upon stress responses elicited by endogenous or exogenous stimuli. The functional role of H3K14 acetylation in response to environmental stress was shown in *S. pombe*, where H3K14R point mutants mimicking hypoacetylated K14 showed elevated sensitivity to stress [41]. In an oxygen–glucose-deprivation model of human SH-SY5Y neuroblastoma cells, HDAC inhibitor treatment led to increased H3K14 and H4K5 acetylation of promoters of *brain-derived neurotrophic factor* (BDNF), a paracrine neuroprotective factor also implicated in HD, and led to elevated BDNF levels and, consequently, increased viability [40]. In U2OS human osteosarcoma cells, PCAF and acetylation of H3K9 and H3K14 at the promoter of the cyclin-dependent kinase inhibitor p21 was found to be required for the p53-dependent transcriptional activation of p21 in response to the p14^ARF^ tumor suppressor, MDM2-p53 interaction inhibitor treatment, and genotoxic stress [39]. H3K14 acetylation also plays a more direct role in the response to genotoxic stress by influencing nucleotide excision repair, a major molecular mechanism responsible for the repair of UV-induced DNA damage. For effective DNA repair, nucleosomes that hinder the access of repair enzymes to DNA must be remodeled. The acetylation of H3K14 does not affect nucleosome stability or unwrapping directly, but it does increase the affinity of nucleosomes for the bromodomain-containing yeast chromatin-remodeling complex RSC (Remodels the Structure of Chromatin) that leads to the more efficient remodeling of nucleosomes and repair of UV-induced pyrimidine dimers [40].

Reduced levels of H3K9/K14 acetylation were also observed under proteopathic stress conditions in various murine models of HD. In the cortex and striatum of N171-82Q and YAC128 HD mice, the levels of bulk histone H3K9/K14 acetylation were found to be reduced, and co-treatment with valproate, an HDAC inhibitor, and lithium, a glycogen synthase kinase 3 (GSK-3) inhibitor, increased H3 acetylation and BDNF and HSP70 expression, and mitigated disease phenotypes [42]. A chromatin immunoprecipitation (ChIP) study of striatal samples of R6/2 mice utilizing a genomic promoter array found that the number of genes with H3K9/K14 acetylation significantly decreased to ~70% of that of the controls. The study found that H3K9/K14 acetylation was associated with an active transcriptional state, but there was no strong correlation between differential transcriptional activity and differential gene acetylation in R6/2 mice, suggesting that acetylation alone was not responsible for the observed gene expression changes [16]. In another study, ChIP-sequencing analysis of hippocampal samples of N171-82Q mice identified a modest, but significant, reduction of acetyl-H3K9/14 and acetyl-H4K12 peaks, and a mild correlation was observed between the transcriptional activity of dysregulated genes and H3K9/14 acetylation levels at the corresponding transcriptional start sites. A small subset of 42 genes, enriched in those coding for Ca^2+^-binding proteins and synapse components, showed both reduced H3K9/14 acetylation and gene expression in N171-82Q hippocampal samples [43]. Similarly, reduced H3K9/14 acetylation at specific gene loci without a change in the bulk acetylation levels was also described in cortico-striatal and cerebellar samples of R6/1 mice [15]. Interestingly, comparative analysis of the set of hypoacetylated genes in the hippocampus of N171-82Q mice and differentially expressed gene data sets of the cerebellum of N171-82Q mice, striatum of R6/2 mice, and caudate nucleus from deceased HD patients identified significant overlaps [15].

To prove that the disturbed acetylation status of H3K14 lysine plays a role in mediating the effects of altered HAT and/or HDAC activity upon mHtt-induced stress, we tested the genetic interactions of *His3.3A-K14* mutant transgenes and loss-of-function mutants of the HAT *Gcn5* and the HDAC *Sirt1* in the HD model. As a subunit of several multiprotein HAT complexes, GCN5 is capable of acetylating the H3K14 residue [41,44,45], and its reduced level was shown to reduce viability and increase neurodegeneration in a fly HD model [27]. Furthermore, reduced GCN5 activity was shown to promote the apoptosis of rat cerebellar granule neurons in a Bim (Bcl-2 Interacting Mediator of cell death)-dependent manner [46]. Sirtuins play intricate, complex roles in HD [47]; in fly models, reduced SIRT1 activity ameliorated disease phenotypes [28,48]. By mutating H3.3K14 lysine to glutamine, we mimicked a constant acetylation state that mitigated the phenotypes of HD flies. We found that this modification was epistatic over *Gcn5,* i.e., the presence of H3.3K14Q prevented the negative effects of reduced *Gcn5* on HD phenotypes. The positive effects of the heterozygous loss of *Sirt1* on HD phenotypes, on the other hand, were only partially averted. These results imply that the disease-modifying effects of GCN5 are primarily mediated via H3K14 acetylation, while in the case of SIRT1, the deacetylation of H3K14 might only partially be responsible for its disease-specific effects.

Thus, we can conclude that the acetylation state of the H3.3K14 residue affects mHtt-induced phenotypes in *Drosophila* suggesting that it might be an important epigenetic contributor to HD pathology. Furthermore, our data suggest that the specific effects of *Gcn5* on mHtt-induced pathology are mediated by the acetylation of this residue.

## 4. Materials and Methods

### 4.1. Drosophila Melanogaster Stocks and Husbandry

To model HD, we used *mHtt* transgenic flies carrying human HTT exon 1 with 120 glutamines (*HTTex1.Q120*) under the control of the yeast upstream activating sequence (UAS). The *w; UAS-HTTex1.Q120* (diseased) and the *w; UAS-HTTex1.Q25* (control) strains [21] were donated by J. Lawrence Marsh (University of California Irvine, USA). *W; P{GAL4-da.G32}UH1* (henceforth da-GAL4)*, P{GawB}elav^C155^* (henceforth *elav-GAL4*), w*; sna^Sco^/CyO; P{tubP-GAL80^ts^}7 (henceforth tubGAL80^ts^), *w^1118^; Gcn5^E333st^ P{w^+mW.hs^ = FRT(w^hs^)}2A e^1^/TM3, P{w^+mC^ = ActGFP}JMR2, Ser^1^*, and *w^1118^; Sirt1^17^/SM6a* lines were obtained from the Bloomington Drosophila Stock Center. The *yw; Df(2L)His3.3A* [49] and *y w M{eGFP.vas-int.Dm}ZH-2A; M{RFP.attP}ZH-86Fb* (ΦX-86Fb) [22] stocks were the generous donations of Konrad Basler (Institute of Molecular Biology, University of Zurich). For our studies, we generated the following strains: *w; UAS-HTTex1.Q120 Df(2L)His3.3A, elav-GAL4; Sb/TM6 Ubx, elav-GAL4; Sp/SM6b, elav-GAL4; tubGAL80^ts^, elav-GAL4; Df(2L)His3.3A; tubGAL80^ts^*, and *w; UAS-HTTex1.Q120 Df(2L)His3.3A; UAS-His3.3A-PTM.* Stocks were maintained and crosses were conducted on standard *Drosophila* medium (3% dry yeast (Busa Kft, Kiskunfélegyháza, Hungary), 4% cornmeal, 2% wheat flour, 9% dextrose (Molar Chemicals, Halásztelek, Hungary), 0.7% agar (Molar Chemicals), and 0.15% Tegosept (Molar Chemicals)) at 18 °C or 25 °C.

### 4.2. Generating H3.3A PTM Mimetic Transgenic Flies

To mimic specific posttranslational modifications of lysine residues of H3.3, first, genomic DNA was prepared from wild-type flies with a NucleoSpin Tissue kit (Macherey-Nagel, Düren, Germany) and the genomic region of *His3.3A* was amplified by PCR using Q5 high-fidelity DNA polymerase (New England Biolabs, Ipswich, MA, USA (NEB)) with primers located upstream (His3.3A_gF) or downstream (His3.3A_gR) of the gene (see Supplementary Table S1 for all of the primer sequences used in the study). The 2174 bp amplicon was inserted into the pJET1.2 vector with a CloneJET PCR cloning kit (Thermo Fisher Scientific, Waltham, MA, USA, (TFS)). This clone was used as the template in PCR to amplify *His3.3A* from the start codon to the last codon before the stop codon using His3.3A_E3C_F (having a KpnI site and an AAA Kozak sequence before the start codon) and His3.3A_E3C_R primers (having an EcoRI site). The resulting amplicon was cut with FastDigest KpnI and EcoRI enzymes (TFS) and cloned to the corresponding sites of the pENTRY3C Gateway entry vector. Site-directed mutagenesis was performed by amplifying the whole pENTRY3C-His3.3A clone in a 22-cycle inverse PCR reaction with Q5 polymerase using primers with base substitutions at the desired positions. PCR amplicons were phosphorylated with T4 polynucleotide kinase (TFS), circularized with T4 DNA ligase (TFS), and treated with DpnI (TFS) to degrade methylated template DNA. Mutated and wild-type His3.3A inserts were subcloned to the pTWF-attB Gateway destination vector, which was modified to carry a φC31 attB sequence (Supplementary Figure S1A). This vector enabled site-directed integration to the genome, GAL4-dependent transcription, and tagging of the expressed protein with a C-terminal FLAG-tag. Using this method, we generated the following mutant histone variant clones: His3.3A-K9Q, His3.3A-K9R, His3.3A-K9M, His3.3A-K14Q, His3.3A-K14R, and His3.3A-K27Q (Supplementary Figure S1B). We generated His3.3A-K27R and His3.3A-K27M previously using the same method [17]. All mutations were verified and validated by Sanger sequencing. These constructs were injected into embryos carrying the *attP-zh86Fb* φC31 docking site to generate transgenic flies by site-specific transgene integration. From individual transformants, homozygous transgenic stocks with the generic genotype of *w; +; UAS-His3.3A-PTM-FLAG* (henceforth *UAS-His3.3A-PTM*) were established. To validate transgenic strains, genomic DNA was prepared from transgenic flies, transgene sequences were PCR-amplified using pTWFattB_Fseq and pTWFattB_Rseq primers, and were then subjected to Sanger sequencing.

### 4.3. Immunoblotting

Transgene expression from the *His3.3A-PTM* constructs was analyzed by immunoblotting. For this, we first crossed *UAS-His3.3A-PTM* transgenic lines with *elav-GAL4* flies to generate *elav-GAL4/+; +; UAS-His3.3A-PTM/+* progenies. Immunoblots were performed on head samples (3 biological replicates per genotype, 40 heads per replicate). Samples were homogenized in sonication buffer (50 mM Tris-HCl pH 7.9, 2 mM EDTA, 50 mM NaCl, 0.5 mM DTT, and 1× Protease inhibitor cocktail set I (Merck, Rahway, NJ, USA)) using a plastic pestle, then denatured by boiling in 2X SDS loading buffer (100 mM Tris-HCl pH 6.8, 200 mM DTT, 4% SDS, 0.2% bromophenol blue, and 20% glycerine) containing 5% β-mercaptoethanol for 5 min. After centrifugation for 10 min at 13,000 RPM, the sample supernatants were loaded in 10% polyacrylamide gel and the proteins were separated by electrophoresis in a discontinuous Tris–tricine–SDS buffer system. After electrotransfer to Amersham Protran Premium 0.45 μm nitrocellulose membrane (GE Healthcare Life Sciences, Chicago, IL, USA), the membranes were blocked in 5% nonfat milk and incubated with the following primary and secondary antibody combinations at the indicated dilutions: mouse monoclonal anti-M2-FLAG (1:5000, F1804, Sigma, Burlington, MA, USA) with rabbit-anti-mouse IgG-HRP (1:10000, P0260, Agilent/Dako, Santa Clara, CA, USA) and rabbit polyclonal anti-H3 (1:4000, ab1791, Abcam, Cambridge, UK) with goat–anti-rabbit IgG-HRP (1:20000, P0448, Agilent/Dako). Immunoblots were developed with Immobilon Western Chemiluminescent HRP substrate (Merck) and imaged with a C-DiGit chemiluminescent blot scanner (Li-Cor Biosciences, Lincoln, NE, USA).

### 4.4. Immunohistochemistry

Transgenic *His3.3A-PTM* lines were crossed with *da-GAL4* to generate a *w; +; da-GAL4/UAS-His3.3A-PTM* progeny. Wandering third instar larvae reared at 25 °C were dissected in 1× PBS and incubated in fixative (4% paraformaldehyde and 0.2% Triton X-100 in 1× PBS) for 20 min. Dissected tissues were washed and permeabilized (0.5% Triton X-100 in 1× PBS) for 20 min, followed by blocking (5% BSA and 0.1% TWEEN-20 in 1× PBS) for 30 min. Samples were incubated with the following primary and secondary antibody combination at the indicated dilutions: mouse monoclonal anti-M2-FLAG (1:750, F1804, Sigma) with goat–anti-mouse GAM-AlexaFluor488 (1:1500, R37120, TFS). After several washing steps, the samples were incubated with DAPI (1:1000, Sigma) for 10 min, followed by a final washing step before dissecting and mounting in Fluoromont (F4680, Sigma). Images were captured with a Nikon Eclipse 80i fluorescent microscope (Nikon, Tokyo, Japan).

### 4.5. Histone Fractionation by Salt Elution

H3.3-PTM histones were fractionated by salt elution as described in [50], with minor modifications. For histone extraction, head samples of 1–3-day-old *elav-GAL4; His3.3A, elav-GAL4; His3.3A-PTM*, and *elav-GAL4* control males were collected and three biological replicates per genotype were analyzed.

One hundred heads were homogenized in 120 μL of Buffer A (0.23 M sucrose, 15 mM Tris–HCl pH 7.5, 60 mM KCl, 15 mM NaCl, 0.15 mM spermine (Sigma), 0.5 mM spermidine (Sigma), 0.2 mM PMSF, 14 mM 2-mercaptoethanol, and 0.25 mM MgCl_2_) with a pestle and then centrifuged (3300× *g*, 15 min, 4 °C). The supernatant (non-chromatin-bound fraction) was transferred to a clean tube. The pellet was washed once with 100 μL of Buffer A and once with 100 μL of Buffer B (15 mM Tris–HCl pH 7.5, 60 mM KCl, 15 mM NaCl, 0.15 mM spermine, 0.5 mM spermidine, 0.2 mM PMSF, 14 mM 2-mercaptoethanol, and 0.25 mM MgCl_2_). After centrifugation (3300× *g*, 15 min, 4 °C), the pelleted sample was resuspended in Buffer B complemented with 0 mM, 100 mM, 500 mM, 800 mM, 1400 mM, or 2000 mM of NaCl and incubated on ice for 10 min with agitation. Samples were centrifuged (3300× *g*, 15 min, 4 °C), the supernatant (salt eluted chromatin-bound fraction) was collected in fresh tubes, and the pellet was redissolved in Buffer A (non-eluted chromatin-bound fraction). For immunoblot analysis, an equal volume of 2× loading buffer (0.125 mM Tris–HCl pH 6.8, 4% SDS, 20% glycerol, 200 mM dithiothreitol, and 0.2% bromophenol blue) was added and the samples were boiled for 10 min. Immunoblots were performed as described above with the following primary and secondary antibody combinations in the indicated dilutions: mouse monoclonal anti-FLAG M2 (1:5000, F3165, Sigma) with rabbit-anti-mouse IgG–HRP (1:5000, P0260, Agilent/Dako); rabbit polyclonal anti-H3 (1:5000, ab1791, Abcam); and rabbit polyclonal anti-H4 (1:1000, ab10158, Abcam) with goat–anti-rabbit IgG-HRP (1:5000, P0448, Agilent/Dako).

### 4.6. Validation of Transgene Expression

The expressions of *UAS-HTTex1.Q120* and *UAS-His3.3A-PTM* transgenes were validated by RT-qPCR. RNA was isolated from 3–5-day-old male heads (at least 3 biological replicates per sampling time with 20 males per replicate) using Trizol Reagent (Invitrogen, Waltham, MA, USA). The RNA concentration and purity were determined by spectrophotometric measurement with a NanoDrop ND-1000 instrument (TFS). After DNaseI (TFS) treatment, the cDNA was prepared from 400 ng of total RNA using TaqMan Reverse Transcription Reagents (TFS) with random hexamer primers following the recommendations of the manufacturer. The resulting cDNA was diluted 1:5 and used for qPCR following the SYBR green method with Luna Universal qPCR Master Mix (NEB) in a PikoReal Real-Time PCR System (TFS) using HttQ120_qF–pUAST_qR and H3.3A_qF–pTWFattB_rseq primer pairs. qPCR data were normalized to *Alpha-tubulin at 84B* (CG1913) housekeeping gene; for statistical analysis, a one-way ANOVA with Tukey’s HSD post hoc test was used.

### 4.7. Drosophila Crosses and Eclosion Analysis

Crosses were conducted at 25 °C unless otherwise noted. To test the effect of *His3.3A-PTM* transgene expression on the viability of wild-type flies, a two-step crossing scheme was performed. First, transgenic *UAS-His3.3A-PTM* lines were crossed with *w; TM3 Sb/TM6 Hu* virgins; then, *w; UAS-His3.3A-PTM/TM3 Sb* male progeny was crossed with *elav-GAL4* virgins (5 females × 5 males, at least 40 individual crosses per genotype), generating progenies of four genotypes: *elav-GAL4/w; UAS-His3.3A-PTM/+, elav-GAL4/w; TM3 Sb/+*, *elav-GAL4/Y; UAS-His3.3A-PTM/+,* and *elav-GAL4/Y; TM3 Sb/+*. The number of flies in each genotype category was counted for 5 consecutive days and viability was expressed as the ratio of eclosed *His3.3A-PTM*-expressing flies to *elav-GAL4* driver-only siblings. At least 90 flies per *His3.3A-PTM* line crosses were counted; for statistical analysis, a one-way ANOVA with Tukey’s HSD post hoc test was used.

To test the effects of neuronal *His3.3A-PTM* transgene expression starting at embryonic development in HD flies, *UAS-His3.3A-PTM* lines were first crossed with *elav-GAL4; +; Sb/TM6Ubx* virgins to generate *elav-GAL4*/Y; *+; UAS-His3.3A-PTM/Sb* male progenies that were crossed with *w; UAS-HTTex1.Q120* virgins (5 females × 5 males, at least 40 individual crosses per genotype), generating progenies of four genotypes: *w*/Y; *UAS-HTTex1.Q120/+; UAS-His3.3A-PTM/+, w*/Y; *UAS-HTTex1.Q120/+; +/Sb, elav-GAL4/w; UAS-HTTex1.Q120/+; UAS-His3.3A-PTM/+*, and *elav-GAL4/w; UAS-HTTex1.Q120/+; +/Sb*. The number of progenies in each genotype category was counted for 5 consecutive days and viability was expressed as the ratio of eclosed *His3.3A-PTM*-expressing HD flies to *His3.3A-PTM*-non-expressing siblings. At least 1200 flies per *His3.3A-PTM* line crosses were counted; for statistical analysis, a one-way ANOVA with Tukey’s HSD post hoc test was used.

To test the effect of neuronal *His3.3A-PTM* expression, starting at the beginning of the adult stage, on HD flies, *w; UAS-HTTex1.Q120 Df(2L)His3.3A; UAS-His3.3A-PTM* males were mated with *elav-GAL4; tubGAL80^ts^* females at 18 °C to generate *elav-GAL4/w; UAS-HTTex1.Q120 Df(2L)His3.3A/+; tubGAL80^ts^/UAS-His3.3A-PTM* progenies raised at 18 °C. Eclosed progenies were transferred to 30 °C to induce GAL4-dependent gene expression.

To test the effects of *Gcn5* and *Sirt1* in HD flies, *w^1118^; Gcn5^E333st^/TM3* males were crossed with *elav-GAL4; Sb/TM6* females, while *w^1118^; Sirt1^17^/SM6a* males were crossed with *elav-GAL4; Sp/SM6b* females. F1 *elav-GAL4/Y; Gcn5^E333st^/TM6* or *elav-GAL4/Y; Sirt1^17^/Sp* male progenies were mated with *w; Df(His3.3A) UAS-HTTex1.Q120; His3.3A-PTM* females (5 females × 5 males, at least 60 individual crosses per genotype), generating progenies of four genotypes: *elav-GAL4/w; Df(His3.3A) UAS-HTTex1.Q120/+; His3.3A-PTM/Gcn5^E333st^, elav-GAL4/w; Df(His3.3A) UAS-HTTex1.Q120/+; His3.3A-PTM/TM6, w/Y; Df(His3.3A) UAS-HTTex1.Q120/+; His3.3A-PTM/Gcn5^E333st^,* and *w/Y; Df(His3.3A) UAS-HTTex1.Q120/+; His3.3A-PTM/TM6* or *elav-GAL4/w; Df(His3.3A) UAS-HTTex1.Q120/Sirt1^17^; His3.3A-PTM/+, elav-GAL4/w; Df(His3.3A) UAS-HTTex1.Q120/Sp; His3.3A-PTM/+, w/Y; Df(His3.3A) UAS-HTTex1.Q120/Sirt1^17^; His3.3A-PTM/+*, and *w/Y; Df(His3.3A) UAS-HTTex1.Q120/Sp; His3.3A-PTM/+*, respectively. After eclosion, the number of progenies in the four genotype categories was counted for 5 consecutive days and viability was expressed as the ratio of eclosed *His3.3A-PTM–Gcn5^E333st^* or *His3.3A-PTM–Sirt1^17^* HD flies to *His3.3A-PTM*-only siblings. At least 1800 flies per *His3.3A-PTM* line crosses were counted; for statistical analysis, a one-way ANOVA with Tukey’s HSD post hoc test was used.

### 4.8. Longevity Analysis

To determine the lifespan of *Drosophila* expressing *mHtt* and *His3.3A-PTM* transgenes from embryogenesis, flies raised at 25 °C were collected in a 24 h period after eclosion and transferred to fresh vials every second day. The number of deceased individuals was recorded daily. At least 150 flies per genotype were used for longevity analysis.

To determine the longevity of HD flies expressing *UAS-His3.3A-PTM* transgenes starting from eclosion, *elav-GAL4/w; UAS-HTTex1.Q120 Df(2L)His3.3A/+; tubGAL80^ts^/UAS-His3.3A-PTM* flies that were raised at 18 °C and eclosed in a 24 h time period were transferred to fresh vials and exposed to 30 °C to induce transgene expression in the adult nervous system. Flies were stored at 30 °C and transferred to fresh vials every second day, and the number of deceased individuals was recorded daily. At least 200 flies per genotype were used for longevity analysis.

To determine the longevity of *Drosophila* co-expressing *UAS-HTTex1.Q120* and *His3.3A-PTM* transgenes and heterozygous for *Gcn5^E333st^* or *Sirt1^17^* alleles, flies raised at 25 °C were collected in a 24 h time period after eclosion and transferred to fresh vials every second day. The number of deceased individuals was recorded daily. At least 100 flies per genotype were used for longevity analysis.

The evaluation of longevity analysis was carried out using OASIS (Online Application and Screening Information System) software [51], and Fisher’s exact test was used for the statistical analysis of the flies’ median lifespans.

### 4.9. Neurodegeneration Measurement

To measure neurodegeneration, a pseudopupil assay [23] was conducted on flies expressing *mHtt* and *His3.3A-PTM* transgenes from embryogenesis. Two-day-old (testing *His3.3A-PTM* lines) or five-day-old (analysis of *Gcn5^E333st^* and *Sirt1^17^* interaction crosses) flies were decapitated and their heads were immobilized on microscopic slides with clear nail polish. The number of intact rhabdomeres per ommatidium in the compound eye was counted under a Nikon Eclipse 80i microscope (Nikon) using a 50× oil-immersion lens. At least 30 ommatidia per eye and at least 10 eyes per treatment were scored and expressed as the mean ± SEM normalized to wild-type *His3.3A*-expressing control; for statistical analysis, a one-way ANOVA with Tukey’s HSD post hoc test was used.

### 4.10. Climbing Assay

The motor activity of HD flies expressing *UAS-His3.3A-PTM* transgenes starting from eclosion was measured by a climbing assay, which is based on the characteristic negative geotaxis of adult *Drosophila*. Age-synchronized 10-day-old adults were transferred to glass vials in cohorts of 20–30 flies, and 5 min long video recordings were obtained of flies climbing upward for 15 s after being tapped down. Motor activity was characterized by the height and speed of climbing between the second and third seconds using Flytracker [21] with manual curation. The motor activity of *His3.3A-PTM*-overexpressing HD flies was measured in 3 separate groups (K9, K14, and K27 lysine mutations), with each group having its own control of non-modified *His3.3A*-overexpressing HD flies; measurements were obtained with at least 3 biological replicates, and for statistical analysis, two-way ANOVA and one-way ANOVA with Tukey’s HSD post hoc test were used.

### 4.11. Activity Recording and Analysis

To test the effects of H3.3-PTM mutations on the daily activity of HD flies expressing *UAS-HTTex1.Q120 elav-GAL4;+; tubGAL80^ts^* virgins were mated with *w; UAS-HTTex1.Q120 Df(2L)His3.3A; UAS-His3.3A-PTM* flies at 18 °C to generate *elav-GAL4/Y; UAS-HTTex1.Q120 Df(2L)His3.3A/+; tubGAL80^ts^/UAS-His3.3A-PTM* progenies. As non-diseased controls, *elav-GAL4*/*Y*; *UAS-HTTex1.Q25 Df(2L)His3.3A*/*+*; *tubGAL80^ts^*/*+* flies were used. PTM males that eclosed in a 24 h time period were transferred to fresh vials and exposed to 30 °C to induce transgene expression. Flies were synchronized and entrained by exposing them to 12:12 h light (~250 lx):dark (LD) cycles for 9 days before performing the activity recordings with a DAM2 Drosophila Activity Monitor (TriKinetics Inc, Waltham, MA, USA) that could record the activity of 32 individual flies simultaneously. The daily activity of *His3.3A-PTM*-overexpressing HD flies was measured in 3 separate groups (K9, K14, and K27 lysine mutations), with each group having its own control of non-modified *His3.3A*-overexpressing HD flies. We recorded the daily activity of 10-day-old flies over a period of 24 h, starting from ZT0 (Zeitgeber Time: refers to the time in hours during a light-dark cycle where ZT0 = lights on and ZT12 = lights off). Data were collected with a DAMSystem3 for Windows (TriKinetics Inc, Waltham, MA, USA) and analyzed using pySolo analysis software [52] and by Excel (Microsoft, Redmond, WA, USA) functions. At least 24 flies per genotype were used for daily activity measurements and all data are presented as the mean ± standard error of the mean (SEM); for statistical analysis, all parameters were compared by Student’s *t*-test or a one-way ANOVA with Tukey’s HSD post hoc test.

## Data Availability

All relevant data are within the manuscript, Supplementary Material, and Supporting Information files. Other datasets used and/or analyzed during the current study are available from the corresponding author on reasonable request.

## Supplementary Materials

The supporting information can be downloaded at: https://www.mdpi.com/article/10.3390/ijms232315173/s1.
