# Supplementary material for: Acetylation State of Lysine 14 of Histone H3.3 Affects Mutant Huntingtin Induced Pathogenesis
Source: Int J Mol Sci. 2022 Dec 2;23(23):15173. doi: 10.3390/ijms232315173 (PMC9738228; doi:10.3390/ijms232315173)
Supplement: Supplementary file 1 [file ijms-23-15173-s001.zip › ijms-2026605-supplementary.pdf]

Supplementary material

Acetylation State of Lysine 14 of Histone H3.3 Affects Mutant Huntingtin Induced Pathogenesis

Anikó Faragó [1,2,#], Nóra Zsindely [1,#], Anita Farkas [1,2], Alexandra Neller [1], Fruzsina Siági [1,2], Márton Richárd Szabó [3,4], Tamás Csont [3,4], László Bodai [1,*]

[1] Department of Biochemistry and Molecular Biology, Faculty of Science and Informatics, University of Sze-ged, H-6726 Szeged, Közép fasor 52. Hungary

[2] Doctoral School in Biology, Faculty of Science and Informatics, University of Szeged, H-6726 Szeged, Hungary

[3] Department of Biochemistry, Albert Szent-Györgyi Medical School, University of Szeged, H-6720 Szeged, Hungary

[4] Interdisciplinary Centre of Excellence, University of Szeged, H-6720 Szeged, Hungary

[#] these authors contributed equally

[*] Correspondence: bodai@bio.u-szeged.hu

Supplementary Figure S1

A

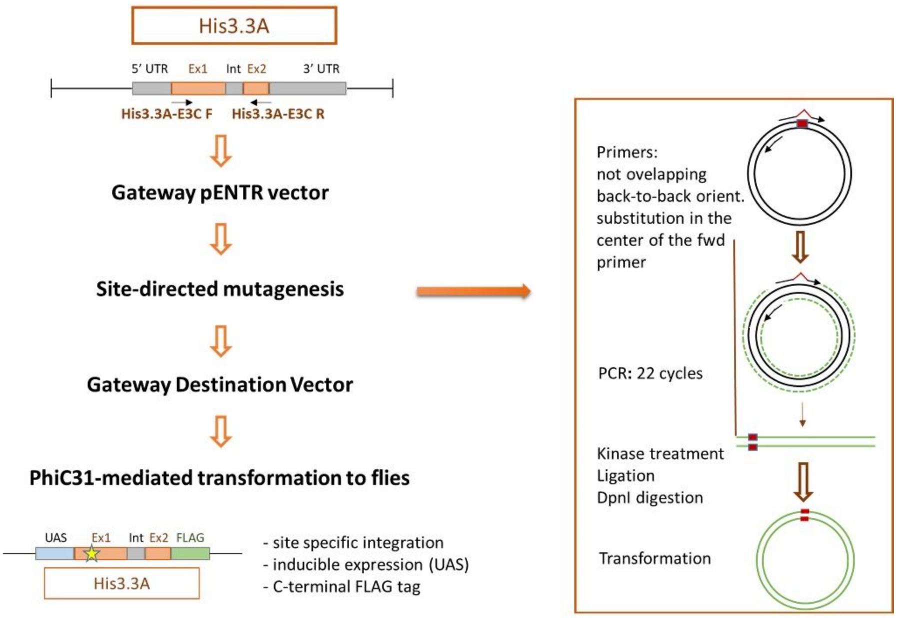

B

		1	2	3	4	5	6	7	8	9	10	11	12	13	14	15	16	17	18	19	20	21	22	23	24	25	26	27	28	29	30
**H3.3A**	M	A	R	T	K	Q	T	A	R	K	S	T	G	G	K	A	P	R	K	Q	L	A	T	K	A	A	R	K	S	A	P
**H3.3A-K9Q**	M	A	R	T	K	Q	T	A	R	Q	S	T	G	G	K	A	P	R	K	Q	L	A	T	K	A	A	R	K	S	A	P
**H3.3A-K9R**	M	A	R	T	K	Q	T	A	R	R	S	T	G	G	K	A	P	R	K	Q	L	A	T	K	A	A	R	K	S	A	P
**H3.3A-K9M**	M	A	R	T	K	Q	T	A	R	M	S	T	G	G	K	A	P	R	K	Q	L	A	T	K	A	A	R	K	S	A	P
**H3.3A-K14Q**	M	A	R	T	K	Q	T	A	R	K	S	T	G	G	Q	A	P	R	K	Q	L	A	T	K	A	A	R	K	S	A	P
**H3.3A-K14R**	M	A	R	T	K	Q	T	A	R	K	S	T	G	G	R	A	P	R	K	Q	L	A	T	K	A	A	R	K	S	A	P
**H3.3A-K27Q**	M	A	R	T	K	Q	T	A	R	K	S	T	G	G	K	A	P	R	K	Q	L	A	T	K	A	A	R	Q	S	A	P
**H3.3A-K27R**	M	A	R	T	K	Q	T	A	R	K	S	T	G	G	K	A	P	R	K	Q	L	A	T	K	A	A	R	R	S	A	P
**H3.3A-K27M**	M	A	R	T	K	Q	T	A	R	K	S	T	G	G	K	A	P	R	K	Q	L	A	T	K	A	A	R	M	S	A	P

Generation of H3.3A PTM mimetic transgenic flies. (A) Genomic *His3.3A* was amplified from the start codon to the last codon before the stop codon. The resulting amplicon was cloned to pENTRY3C Gateway entry vector. Site-directed mutagenesis was performed by amplifying the whole pENTRY3C-His3.3A clone in a 22-cycle inverse PCR reaction using primers having base substitutions at the desired positions (Supplementary Table S1). PCR amplicons were phosphorylated with T4 polynucleotide kinase, circularized with T4 DNA ligase, and treated with DpnI to degrade methylated template DNA. Mutated and wild-type H3.3A inserts were subcloned to pTWF-attB Gateway destination vector that was modified to carry a φC31 attB sequence. This vector enables site-directed integration, GAL4-dependent transcription, and tagging of the expressed protein with a C-terminal FLAG-tag. (**B)** Amino acid sequences of N-terminal tails of wild-type and H3.3A-PTM mimetic mutant histones.

Supplementary Figure S2

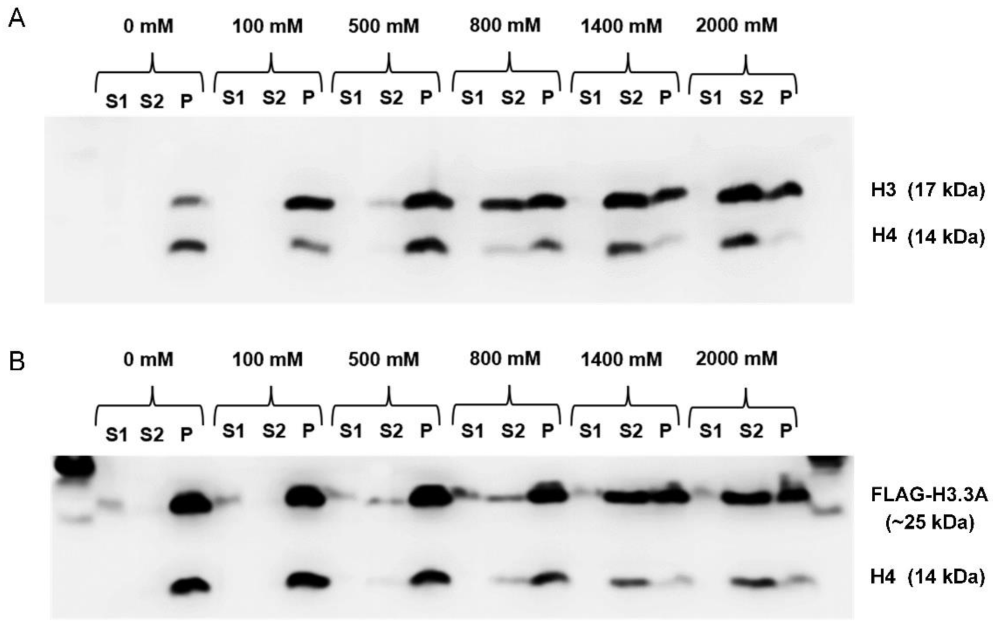

Immunoblot analysis of histone salt elution experiments on head samples of flies overexpressing His3.3A. Anti-H3 (A) and anti-FLAG (B) antibody was used to detect endogenous H3 and transgenic His3.3-FLAG proteins, respectively. Anti-H4 antibody was used as control. 0-2000 mM NaCl was used to optimize salt concentration for H3.3 elution from chromatin. Endogenous H3 does not appear in the cytosolic supernatant (S1), however, due to overexpression negligible transgenic H3.3 can be detected in this fraction. Upon increasing salt concentration histones are eluated and appear in the nuclear supernatant (S2), but it is important to note that a substantial amount still remains in the nuclear pellet (P) indicating that it is chromatin-bound. Using 800 mM NaCl notable amount of endogenous H3 appear in S2 fraction, while in the case of transgenic H3.3 only the use of 1400 mM salt can induce the same elution rate, suggesting that H3.3 binds stronger to chromatin. Thus, to investigate H3.3 PTM mimetic mutants we used 1400 mM NaCl in further histone salt elution studies.

Supplementary Figure S3

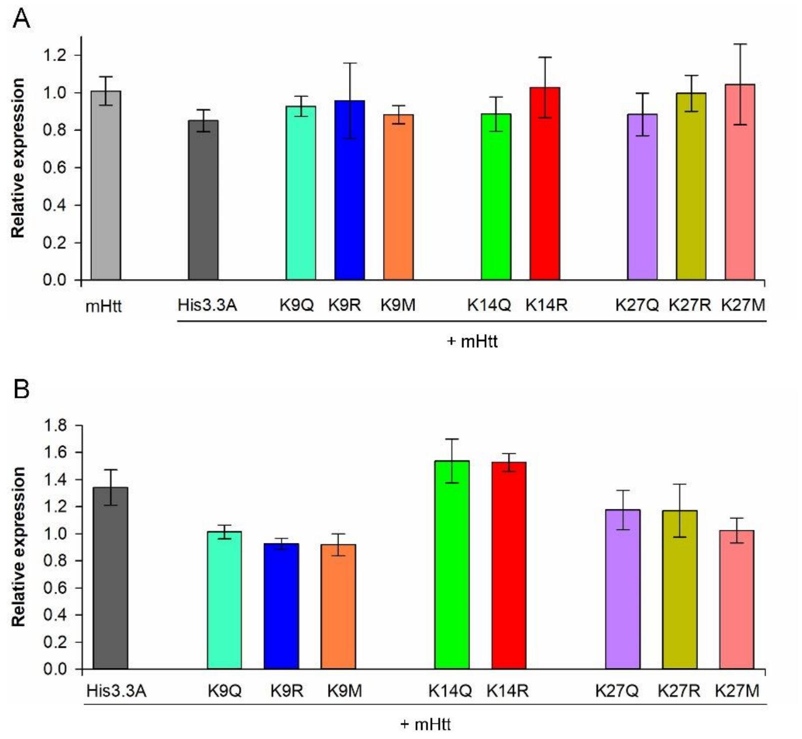

The transcript level of *mHtt* and *His3.3A-PTM* transgenes did not change if co-expressed with the elav-GAL4 driver. (A) Relative expression of *mHtt* transgene does not change significantly (ANOVA) if co-expressed with *His3.3A* or *His3.3A-PTM* transgenes compared to the *mHtt* only control. (**B)** Relative expression of *His3.3A-PTM* transgenes does not change significantly (ANOVA) if co-expressed with *mHtt* transgenes compared to the *His3.3A* expressing control. The gene expression levels of mutants of the same position are similar, however, non-significant differences in the expression profiles of lysine modifications in the different positions can be observed.

Supplementary Figure S4

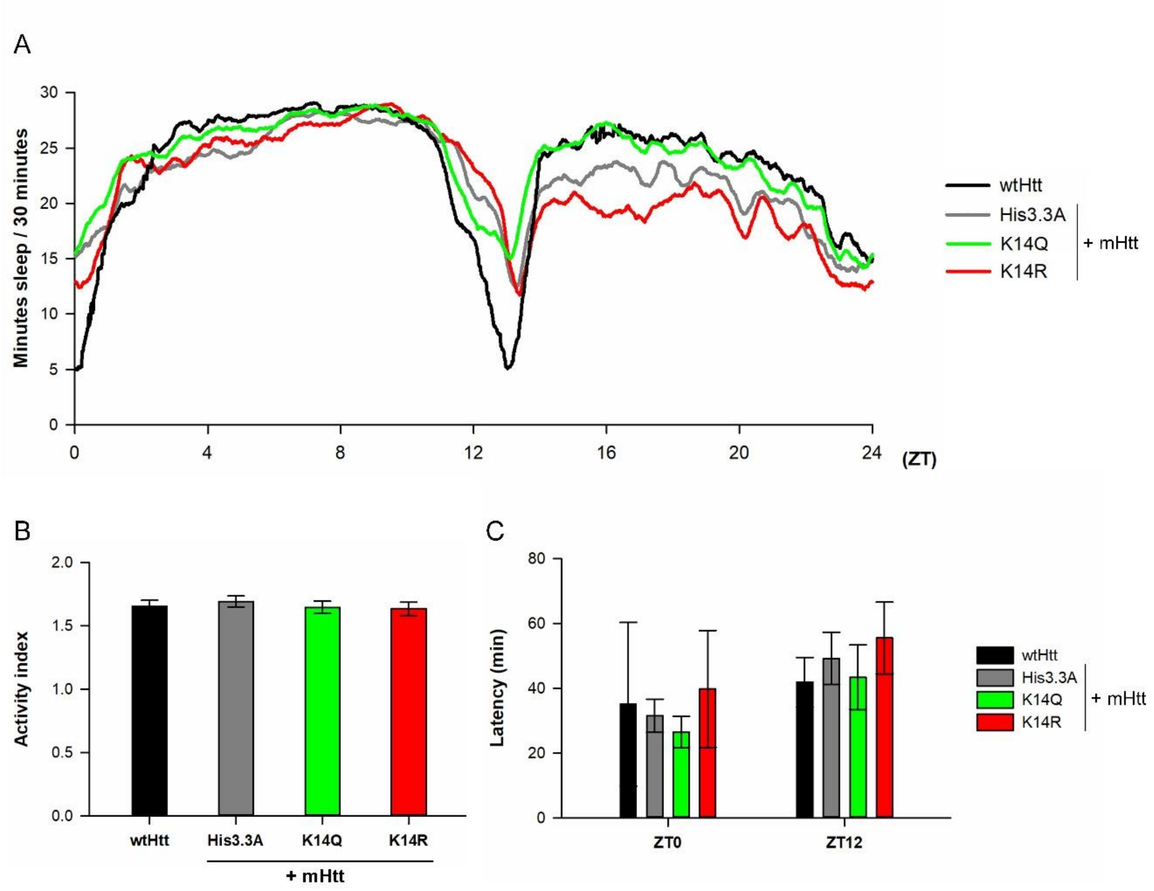

Sleep phenotypes of flies expressing *wtHtt* or co-expressing *mHtt* + *His3.3A*, *His3.3A-K14Q* or *His3.3A-K14R* transgenes. **(A)** Sleep patterns of male flies expressing *wtHtt* or co-expressing *mHtt* + *His3.3A* transgenes in the adult nervous system under the influence of *elav-GAL4*; *tubGAL80*^{ts} with heterozygous *His3.3A*^{KO} background. Male flies co-expressing *mHtt* + *His3.3A* showed hyperactivity and spent less time asleep during nighttime compared to *wtHtt* expressing healthy flies. The expression of *His3.3A-K14Q* suppressed, while that of *His3.3A-K14R* enhanced the disturbed sleep pattern of HD flies. **(B)** Activity index – movement count normalized to the time spent awake - of male flies expressing *wtHtt* or co-expressing *mHtt* + *His3.3A* transgenes in the adult nervous system under the influence of *elav-GAL4*; *tubGAL80*^{ts} with heterozygous *His3.3A*^{KO} background. The activity index is similar in all genotypes, indicating that the observed hyperactivity of HD flies is the consequence of reduced sleep instead of increased motor activity. **(C)** Sleep-onset latency - time between lights ON/OFF and the start of the first sleep episode – of male flies expressing *wtHtt* or co-expressing *mHtt* + *His3.3A* transgenes in the adult nervous system under the influence of *elav-GAL4*; *tubGAL80*^{ts} with heterozygous *His3.3A*^{KO} background. We could not detect significant differences in the sleep-onset latency between the different genotypes.

Supplementary Figure S5

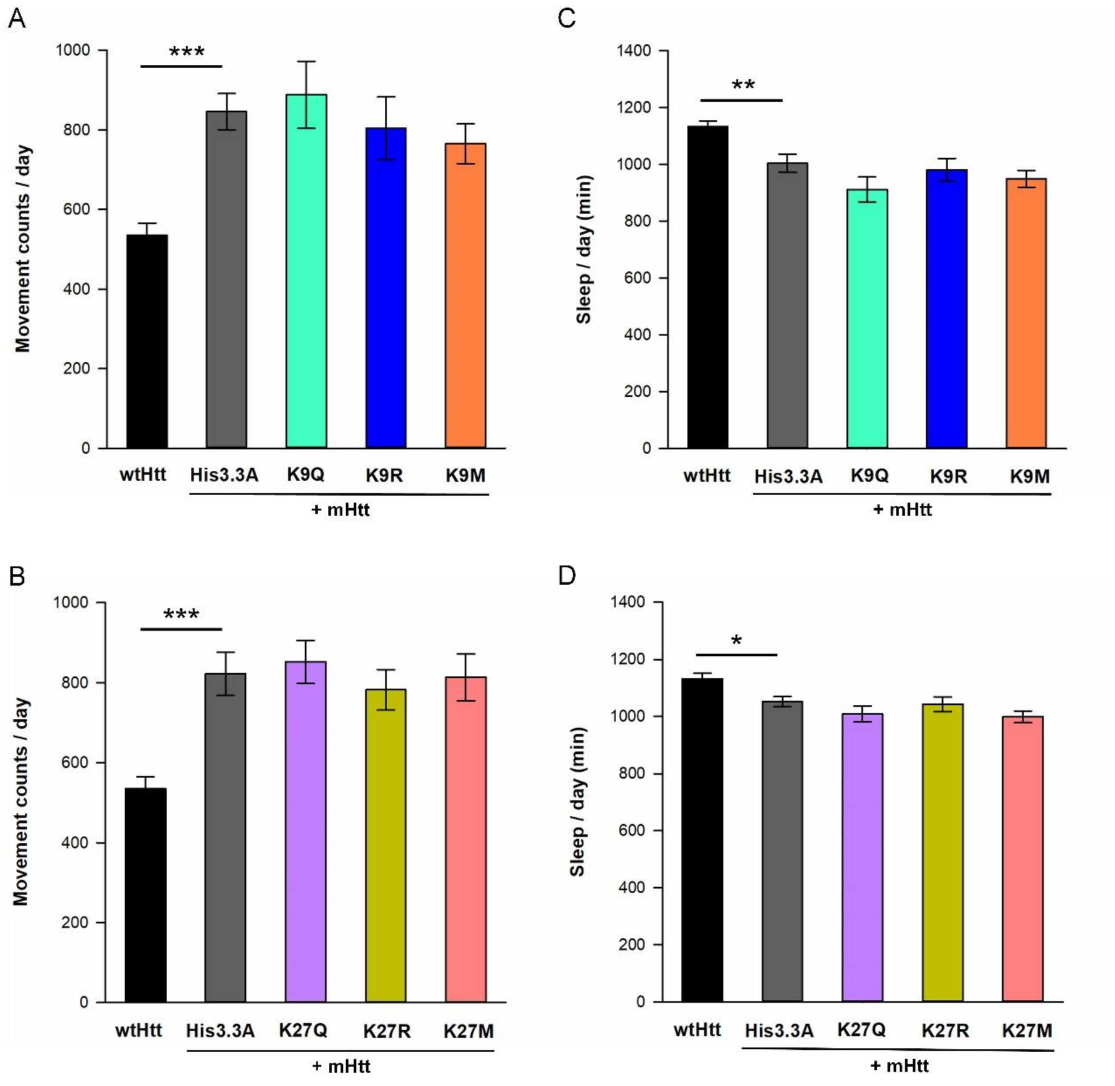

There are no significant differences in daily movement counts and length of daily sleep in HD flies expressing *His3.3A-K9* or *His3.3A-K27* mutant transgenes. (A,B) Total daily movement counts of male flies expressing *wtHtt* or co-expressing *mHtt* + *His3.3A* transgenes in the adult nervous system under the influence of *elav-GAL4*; *tubGAL80*^{ts} with heterozygous *His3.3A*^{KO} background. The graphs show the average of the total number of movements during a 24h time period, error bars represent standard error. ** P≤0.01, *** P≤0.001, ANOVA. **(C,D)** Total amount of daily sleep of male flies expressing *wtHtt* or co-expressing *mHtt* + *His3.3A* transgenes in the adult nervous system under the influence of *elav-GAL4*; *tubGAL80*^{ts} with heterozygous *His3.3A*^{KO} background. The graphs show the average total amount of sleep in minutes during a 24h time period, error bars represent standard error. * P≤0.05, *** P≤0.001, ANOVA.

Supplementary Table S1

Primer name	5'-3' sequence
His3.3A_gF	ACTTCCTCTTCTCCATACCC
His3.3A_gR	ACCCATAAATTCCCTGACA
His3.3A_E3C_F	GGTACCAAAATGGCACGTACCAAGC
His3.3A_E3C_R	GAATTCGCGGCCCGCTCGCCACG
K9Q_F	GCAAACAGCCCGTCAATCGACCGGAGG
K9Q_R	CCTCCGGTCGATTGACGGGCTGTTTGC
K9R_F	GCCGCTAGATCGACCGGAGGC
K9M_F	GCCCGTATGTCGACCGGAGGC
K9R/M_R	TGTTTGCTTGGTACGTGCCAT
K14_F	CGCAAGCAGCTGGCCACCAAG
K14Q_R	GGGCGCCTGGCCTCCGGTCGA
K14R_R	GGGCGCGCGGCCTCCGGTCGA
K27_F	CCATCCACCGGCGGAGTGAAG
K27M_R	CGCCGACATACGGGCCGCCTT
K27R_R	CGCCGAGCGACGGGCCGCCTT
pTWFattB_Fseq	GCAACTACTGAAATCTGCCAAGAAG
pTWFattB_Rseq	AAGGTTCCTTCACAAAGATCCTCT
HttQ120_qF	GCCTCTGCACCGACCATAA
pUAST_qR	TGGAACTGATGAATGGGAGCA
H3.3A_qF	TTGGAGCCCTACAGGAGGC

The sequences of oligonucleotide primers used in the study. Base substitutions in primers used for *in vitro* mutagenesis are underlined.
